# Supplementary material for: Service utilization and HIV outcomes among transgender women receiving Ryan White Part A services in New York City
Source: PLoS One. 2021 Jul 1;16(7):e0253444. doi: 10.1371/journal.pone.0253444 (PMC8248705; doi:10.1371/journal.pone.0253444)
Supplement: Attachment — Submitted filename: Response_to_Reviewers.doc [file pone.0253444.s001.doc]

**Jacinthe A. Thomas, MPH**

Senior Research Analyst

Bureau of HIV

nyc.gov/health

**42-09 28^th^ Street, 22^nd^ Floor**

**Queens, NY 11101**

January 22, 2021

Dear Dr. Morgan,

My co-authors and I are pleased at the opportunity to revise and resubmit our manuscript entitled ‘Service utilization and HIV outcomes among transgender women receiving Ryan White Part A services in New York City’ (PONE-S-20-38897).

We are grateful for your constructive comments and suggestions on our manuscript. We have carefully considered all the comments, and respond to each critical point below, in blue, noting where revisions have been made to the manuscript. We explained why no participant consents were required for this retrospective analysis, updated Tables 2 and 3 to reflect the p-values according to PLOS ONE’s style requirements, and included the new results using cisgender women instead of cisgender men as the reference group in Table 5.

We have uploaded both a clean and a tracked version of the revised manuscript.

**Responses to reviewers’ comments**

1. Please provide additional details regarding participant consent. In the ethics statement in the Methods and online submission information, please ensure that you have specified (1) whether consent was informed and (2) what type you obtained (for instance, written or verbal, and if verbal, how it was documented and witnessed). If your study included minors, state whether you obtained consent from parents or guardians. If the need for consent was waived by the ethics committee, please include this information.

If you are reporting a retrospective study of medical records or archived samples, please ensure that you have discussed whether all data were fully anonymized before you accessed them and/or whether the IRB or ethics committee waived the requirement for informed consent. If patients provided informed written consent to have data from their medical records used in research, please include this information.

Response: No participant consents were required for this retrospective analysis, which utilized secondary data that have been reported to the NYC Health Department by our Ryan White Part A (RWPA)-funded service provider agencies and by HIV medical care providers and laboratories, as mandated by NYS laws. Both the RWPA and surveillance data sets that were used were fully de-identified prior to analysis. Only designated staff members who have undergone confidentiality training have access to these data sets. All designated staff members are expected to adhere to the Department of Health data security and confidentiality protocols. This analysis plan was reviewed by the NYC Department of Health IRB and was categorized as public health surveillance, not human subjects research. As a result, no informed consent requirement applies.

2. We note that you have indicated that data from this study are available upon request. PLOS only allows data to be available upon request if there are legal or ethical restrictions on sharing data publicly. For information on unacceptable data access restrictions, please see [http://journals.plos.org/plosone/s/data-availability#loc-unacceptable-data-access-restrictions](http://journals.plos.org/plosone/s/data-availability#loc-unacceptable-data-access-restrictions).

In your revised cover letter, please address the following prompts:

a) If there are ethical or legal restrictions on sharing a de-identified data set, please explain them in detail (e.g., data contain potentially identifying or sensitive patient information) and who has imposed them (e.g., an ethics committee). Please also provide contact information for a data access committee, ethics committee, or other institutional body to which data requests may be sent.

Response: Due to legal restrictions (under New York Public Health Law Article 21, Title III) and the confidential nature of HIV surveillance data in New York, public health authorities in New York City cannot release individual-level data on reported HIV cases for purposes other than ensuring appropriate HIV care. This restriction applies even to de-identified patient-level datasets. However, NYC DOHMH staff are available to assist external researchers who may have further specific data questions or uses. An email can be sent to [hivreport@health.nyc.gov](mailto:hivreport@health.nyc.gov) with questions or requests for additional information, which will be answered promptly by NYC DOHMH staff.

**Review Comments to the Author**
1. It doesn’t seem like the authors conducted post-hoc tests to assess differences between their three groups of interest (trans women, ciswomen, and cismen) although they report the results as if they did. The chi-square test only assesses whether two variables are significantly associated, but it doesn’t tell which of the subgroups differ significantly from one another. For example, if percentages for group a, b, and c are 16, 20, and 24% respectively, it is possible that group b is not significantly different from either group a or c, and that the only significant difference is between a and c. In this case, we could report that group a was significantly less like to XXX than group c, but not different from group b. To obtain such details, typically a post-hoc z test with p values corrected with the Bonferroni method is used (which is only a matter a checking a few extra boxes for SPSS). The authors should add a column to their tables reporting which group differences were significant (e.g., a)

Response: Recently, experts have recommended moving away from statistical testing to estimation (Amrhein V, Greenland S, McShane B. Retire statistical significance. Nature 2019;567:305-307.; Greenland S, Senn SJ, Rothman KJ, et al. Statistical tests, P values, confidence intervals and power: a guide to misinterpretations. Eur J Epidemiol 2016;31:337-350.; Wasserstein R, Lazar NA. The ASA statement on p-values: context, process, and purpose. Am Stat 2016;70:129-133.). Following their recommendations, we draw our conclusions of differences across groups based on the effect size, i.e., prevalence ratios, and confidence intervals, not the overall p-values or the p-values from pairwise comparisons with Bonferroni adjustment. We present the overall p-value as a statistical summary of the compatibility between the observed data and what we would expect to see if there were no differences.

2. As per guidelines for the journal (which are the standard for most journals), please report test statistics in the tables (i.e., chi-square) and p values to no less than <.001. <https://journals.plos.org/plosone/s/submission-guidelines>

Response: In the revised manuscript, we have updated Tables 2 and 3 according to the PLOS ONE guidelines, which state the following on p-values: “Report exact p-values for all values greater than or equal to 0.001. P-values less than 0.001 may be expressed as p < 0.001, or as exponentials in studies of genetic associations.”

3. I’m not fully convinced by how the authors defined and operationalized “need.” For instance, “harm reduction need” was defined as “recent” substance use (page 8; also please define “recent”). Is it fair to conclude that anyone who has recently used certain substances has a need for harm reduction? In any case, I think it would be better to report the variable as what it actually measured, that is, substance use. In this case, it would be more accurate to report that X% of participants used substances recently and only Y% of them had accessed harm-reduction services.

Response: The definition that we applied matches the eligibility criteria for RWPA Harm Reduction programs. We recognize that it is not a perfect measure for assessing individual need for substance use-related services. Given that we cannot obtain a more nuanced assessment of problem substance use from routine RWPA provider reporting, we treated ‘hard drug’ use (defined as use of cocaine/crack, heroin, crystal meth, or prescription drugs to get high in the past 3 months) as a proxy for need, leveraging existing data. We have replaced the term ‘recent’ in the revised manuscript, in favor of specifying the 3-month timeframe (line 166, page 8). We limited the measure of need to focus on hard drugs because of their association with dependency or other harms to health and safety. We did not include all substance use (e.g., marijuana use or alcohol use), out of recognition that some substances may be used recreationally with moderation, such that reporting some use does not indicate a need for services to reduce use or reduce the harms from use. Furthermore, based on prior analyses in NYC RWPA programs, hard drug use has been found to be associated with worse health outcomes (Feldman MB, Kepler KL, Irvine MK, Thomas JA. Associations between drug use patterns and viral load suppression among HIV-positive individuals who use support services in New York City. Drug and Alcohol Dependence. 2019; 197:15-21.). As mentioned in the paper, there are some limitations related to the substance use data available for analysis. In the RWPA program, substance use is assessed via self-report, and therefore the data can be subject to social desirability bias, which would result in the under-ascertainment of drug use.

4. The definition of “food and nutrition need” also seemed problematic, although the authors recognized that in the Discussion (page 13). I don’t know that it’s accurate to say that everyone under a certain income level has food and nutrition needs. If the authors have a strong rationale for doing so, they should explain it. Otherwise, they may reconsider their operationalization of “need.”

Response: For clients who were not assessed for food and nutrition need, we based the definition on the national cutoff for SNAP eligibility (130% of the Federal Poverty Level), due to the prevalence of food insufficiency among low-income individuals affected by HIV (Pellowski JA, Kalichman SC, Matthews KA, et al. A pandemic of the poor: social disadvantage and the U.S. HIV epidemic. Am Psychol. 2013;68:197–209.). While we agree that not all clients with that income level have food and nutrition service need, given the cost of living in NYC, people at or below that income level in NYC are likely to experience food insufficiency. In addition, households with incomes below 185% of the poverty threshold (34.3% of all U.S. households) have been found to have higher rates of food insufficiency than the national average, regardless of HIV status (Coleman-Jensen A, Nord M, Singh A. Household Food Security in the United States in 2012, ERR-155, Washington, DC: U.S. Department of Agriculture, Economic Research Service; 2013.).

5. The results report on service “need” and utilization, and then on HIV outcomes; however, there is no connection between the two areas (services and HIV outcomes). In the discussion, the authors explain that poorer HIV outcomes among trans women might be due to less service utilization. Why wasn’t service need or utilization included as a potential predictor of HIV outcomes?

Response: We cannot make that conclusion based on the analyses that were performed for this manuscript. It would have required analyses beyond the scope of this paper, such as looking at doses of services received and change in the outcomes over time (following initiation of services), in order to understand the relationship between service utilization and health outcomes. Also, the RWPA services on which we have data are not the only relevant services a client may be receiving. For example, services funded by Medicaid, Medicare, the Veteran's Administration, and other funding streams outside of RWPA were not available for our analysis. Therefore, we didn’t look at service need or utilization as a potential predictor of HIV outcomes. However, in the discussion, we suggested a potential link between service utilization and outcomes based on the literature, and not based on the specific analyses presented in the paper.

6. Looking at HIV outcomes, the authors did multivariable analyses controlling for age, race/ethnicity, and country of birth. However, there is no mention of bivariate tests to determine which control variables to include. It would be appropriate to report what tests were done to establish why these three control variables were selected. The results of bivariate analyses could be reported as supplemental material, if space is a concern.

Response: We selected age, race/ethnicity, and country of birth as covariates using a directed acyclic graph, by following guidance for authors from editors of respiratory, sleep, and critical care journals (Lederer D, Bell S, Branson R, et al. Control of confounding and reporting of results in causal inference studies. Guidance for authors from editors of respiratory, sleep, and critical journals. Ann Am Thorac Soc 2019;16:22-28.). A previous study has found that bivariable analysis may not be appropriate to screen risk factors to be included in multivariable analysis (Sun G, Shook TL, Kay GL. Inappropriate use of bivariable analysis to screen risk factors for use in multivariable analysis. J Clin Epidemiol 1996;49:907-916.).

7. In table 5, because the confidence interval for durable viral suppression includes 1, I don’t think it’s appropriate to report as statistically significant. It seems like trans women were not significantly different from cis men with regards to durable viral suppression. However, cis women were significantly more likely to present durable viral suppression than cis men (probably compared to trans women as well). Why were cis men chosen as the reference group? In any case, this result and associated conclusions should be revisited.

Response: We agree that cisgender women should be the reference group. We have included the new results using cisgender women as the reference group in Table 5 and updated the manuscript where applicable. The new Table 5 shows that, compared with cisgender women, transgender women were 20% (aPR = 0.80; 95% CI: 0.69, 0.94) less likely to have durable viral suppression, and the confidence interval does not contain 1.

8. On page 7, line 143, “Employment status was categorized as “employed” (for full-time or part-time employment); “unemployed” (for unemployed or unpaid volunteer/peer worker status); and “out of workforce” (for student, retired or homemaker status).” In which category was put a student who is also employed?

Response: All clients reporting any form of paid employment, including students, were categorized as employed. We have clarified the language in the revised manuscript (line 143, page 7) to indicate that the levels of employment, collected during assessment, are mutually exclusive.

9. Page 12, line 223, the authors mention the “ART use measures”. Which ones of the variables are referred to as being about ART use? ART prescription and viral suppression don’t clearly measure use of medication (which sounds more like adherence).

Response: We are referring to ART prescription, derived from the question ‘Is the client currently prescribed ART?’. We have changed the wording/labeling in the revised paper accordingly to match to what is being collected from RWPA enrollees.

10. Were there participants who did not fit in the three sex/gender categories examined, for instance transgender men or nonbinary individuals? If so, please explain the decision to exclude from the analysis and how many were excluded.

Response: We used two variables, sex assigned at birth and gender, to define the three groups. There were 31 transgender men and 3 non-binary individuals who met the overall eligibility criteria but were excluded from the analysis. Transgender men and non-binary individuals were not included as separate groups because of their small numbers in the client cohort available for analysis.

Reviewer #2: 
1. In the introduction, I found myself wondering whether there were also disparities by race/ethnicity? This isn’t key to the article so don’t feel the need to add it, but a sentence may just help set the context more.

Response: This is a great point. However, we did not expect to detect conventional disparities by race/ethnicity, mainly because the overwhelming majority of clients in the NYC RWPA program and in this particular cohort identify as Black and Latinx. For example, overall, 13,196 RWPA clients (88%) and 414 transgender women (91%) included in this analysis were Black or Latinx.

2. Are participants compensated for their time at all? I don’t think so since these are services under RWPA activities but it may make sense to state this clearly. Up to the authors.

Response: No, they are not compensated. There was no time requirement for inclusion in the analysis, since we used existing data sets produced as part of routine reporting on HIV services and HIV laboratory monitoring.

3. Table 1 is missing p-values or, at a minimum, any indication of significance. Please add these.

Response: We understand p-values as measures for inferential purposes, not descriptive ones. Therefore, we did not include p-values in Table 1, but did include them in other tables when we compared outcomes across groups. (Turkiewicz A, Luta G, Hughes HV. Statistical mistakes and how to avoid them -- lessons learned from the reproducibility crisis. Osteoarthritis Cartilage 2018;26:1409e1411.). We will add the p-values to Table 1, if it is a PLOS ONE requirement.

4. Second and third paragraphs of results are missing any mention of these results being from Tables 2 and 3.

Response: In the revised manuscript, we have indicated in those paragraphs which results are from Tables 2 and 3.

Sincerely,

Jacinthe Thomas (for the authors)

Senior Research Analyst, Care & Treatment Research & Evaluation Unit

Bureau of HIV, New York City Department of Health & Mental Hygiene

Phone: (917) 648-2898

E-mail: [jthomas1@health.nyc.gov](mailto:jthomas1@health.nyc.gov)
